# Learnings from a hybrid communities of practice group therapy model to support psilocybin-assisted therapy for patients with a terminal diagnosis

**DOI:** 10.3389/fpubh.2026.1767215

**Published:** 2026-07-20

**Authors:** Vivian WL Tsang, Pamela Kryskow, Crosbie Watler, Pearl Allard, Shannon Dames

**Affiliations:** 1 Department of Continuing Education, Evidence Based Health Care, University of Oxford, Oxford, United Kingdom; 2 Department of Psychiatry, Faculty of Medicine, University of British Columbia, Vancouver, BC, Canada; 3 Department of Family Medicine, Faculty of Medicine, University of British Columbia, Vancouver, BC, Canada; 4 Island Health Services, Nanaimo, BC, Canada; 5 Department of Health and Human Services, Vancouver Island University, Nanaimo, BC, Canada

**Keywords:** end of life distress, existential distress, group therapy, palliative, psilocybin, psychedelic therapy, terminal illness

## Abstract

**Background:**

Psilocybin-assisted therapy is continuing to show significant positive effects for palliative cancer patients, both in reducing symptoms and improving one’s sense of hope and meaning. Most research has focused on individual therapy models, limiting access and overlooking potential community-based benefits.

**Methods:**

This qualitative study describes the results of patients experiencing distress related to a terminal health condition who enrolled in six to eight once-weekly group resilience-based community of practice (CoP) sessions combined with one psilocybin-assisted therapy (PaT) session. This virtual hybrid group therapy model is research informed, with a curriculum that provides knowledge-based content, combined with the relational elements necessary to successfully deliver group-administered psilocybin-assisted therapy. Patients were interviewed using semi-structured question list and transcripts were coded and themes captured in an iterative manner. The CoP sequence included preparation meetings before the in-person medicine session and integration meetings afterward, allowing the group relationship and safety practices to be established before dosing.

**Results:**

Thirty-one PaT sessions were provided for 25 participants within four iterative cohorts over the span of 1 year (November 2021–November 2022). Completion rates were high (84%), with participants reporting enhanced trust in self, improved outlook on life, and strengthened connection to community. Peer support, relational safety, and regulation practices were central to outcomes.

**Conclusion:**

This study demonstrates the feasibility, safety, and psychosocial benefits of group-administered PaT supported by virtual CoPs, providing a scalable, cost-effective model for public health interventions in end-of-life care. Its novelty lies in combining group therapy, hybrid delivery, and resilience-based curriculum to expand access and enhance community support for a vulnerable population. Participants’ mixed feedback also suggests that hybrid group PaT may offer distinctive benefits of peer witnessing and community continuity while requiring careful attention to individual preference, room logistics, and sensory control during group dosing.

## Introduction

Patients diagnosed with a life limiting disease often experience hopelessness, a loss of meaning and purpose, uncertainty, anxiety, and depression (1, 2). While antidepressants and anxiolytics can reduce symptom intensity, they do not address the root causes of mental distress (3, 4).

As psychedelic medicines re-emerge in modern medicine, research is showing significant and enduring positive effects from psilocybin-assisted therapy (PaT) for palliative cancer patients, both in reducing symptoms and improving one’s sense of hope and meaning (5–8). Psilocybin has an established safety profile (9) and is non-addictive (10). These are important considerations when working with vulnerable patients. It has targeted effects on 5-HT2A receptors in the brain, inducing a non-ordinary state of consciousness, enabling a reorientation to difficult life events and an opportunity to notice and deconstruct negative thought patterns (11, 12).

Currently, many studies on psychedelic-assisted therapy use a therapist dyad structure, where a pair of therapists supports one individual (5–8). However, even though the use of psychedelics in group settings is not new, there are few published studies which have looked at this process. Spiritual communities of practice have long been a source of healing among Indigenous peoples, using ayahuasca in South America, peyote in North America, and psilocybin in Mexico (13, 14). Contemporary group-oriented psychedelic work has also included group preparation and/or integration models, including studies in demoralized AIDS survivors (15, 16) and psilocybin combined with meditation and spiritual practices, which included structured support and group integration elements (17). Holotropic Breathwork, developed by Stanislav and Christina Grof, uses breathwork to induce non-ordinary states of consciousness with groups as large as several hundred (18). When aligned with best practices, group treatment models can be effective and safe, while supporting greater access. For example, supportive-expressive group therapy demonstrates effectiveness for terminal cancer patients (19–23). Studies on group therapy show increased emotional expressiveness, enhanced communication skills, and a greater ability to identify barriers that are preventing participants from living life fully in the face of death (19).

Research-informed psychedelic assisted group therapy models are generally lacking. In this study, group therapy is defined as inclusive of group preparation, medicine session, and integration therapy where all therapy components of the treatment process are experienced in a cohort system. This contrasts with programs that dose psychedelic medicines in a group with integration or preparation sessions for participants individually. In a recent review of the literature (24), a study with a cohort of demoralised AIDS survivors utilized a combination of group preparation and integration therapy with one individual psilocybin session (15, 16). A research letter describes a study treating cohorts of three to four patients receiving group therapy for pre-sessions with integration and psilocybin medicine sessions in adjacent rooms open to a common space (25).

To help fill this gap in the literature, this study hopes to contribute to the larger healthcare learning system surrounding psilocybin-assisted therapy in Canada and internationally. This qualitative study describes the results of patients experiencing distress related to a terminal health condition who enrolled in six to eight once-weekly group CoP sessions combined with one group psilocybin medicine session.

## Methods

This program followed an established psychedelic therapy program at Roots to Thrive (RTT), which evolved from a doctoral thesis that focussed on core resilience factors required to address burnout and promote resilience in healthcare providers working in trauma-laden environments (26). It was then adapted to support a 12-week group-administered ketamine-assisted therapy program (KaT) for those with treatment resistant mental health conditions (27). RTT and KaT informed the PaT Communities of Practice (CoP) curriculum, supporting participants with end-of-life distress in an accelerated program. The core practices in the program focus on cultivating awareness of body sensations, emotions, thoughts, values, and relational patterns; regulating the nervous system through breathwork, grounding, tapping/Emotional Freedom Techniques, mindfulness, and relational co-regulation; giving and receiving unconditional positive regard; and clarifying the values, relationships, unfinished conversations, and sources of meaning that participants wished to prioritize near the end of life (28). The curriculum also explicitly oriented participants to the principle of trusting one’s inner healing intelligence, a concept used in psychedelic-assisted therapy preparation to encourage participants to remain open to emergent material without over-directing the medicine session (29, 30). The heterogeneous group model supported the development of a comprehensive multidisciplinary program, incorporating multiple ways of knowing, including biomedical palliative care, nursing, trauma-informed practice, somatic and mindfulness-based approaches, peer learning, reflective writing, and lived experience. This virtual hybrid group therapy model used for preparation and integration was research informed, with two-hour weekly curriculum for 6 to 8 weeks that provides knowledge-based content, combined with the relational elements necessary to successfully deliver one eight-hour group-administered in-person psilocybin medicine session.

The in-person psilocybin medicine session took place in an approximately 1,000 square foot dosing room. Each cohort included up to eight participants. Two therapists/facilitators were present per participant during the medicine session; these individuals could include nurses, therapists, or energy workers, depending on participant and cohort needs. In addition, one physician was present and available during the session, and at least two nurses were present for each group of eight participants. Facilitators remained available to provide therapeutic, somatic, nursing, and medical support according to their roles and participant needs. When participants left the main dosing room for a bathroom break, a rest break, or additional support, a facilitator accompanied them. Separate private or quiet spaces were available for participants who needed reduced stimulation, privacy, or additional one-to-one support during the session.

Patients were recruited from the database of patients who had gone through RTT PaT. These patients were identified as individuals whose depression, anxiety, demoralization, or existential distress was triggered or exacerbated by a terminal cancer diagnosis and who met the full inclusion and exclusion criteria of RTT PaT (Box 1). Participant intentions were formally collected during intake. Participants commonly sought relief from cancer-related distress, support in facing death and loss, a sense of community with others facing similar illnesses, and access to psilocybin therapy in a legally sanctioned and clinically supported environment. The first two cohorts of patients were enrolled in the study through Canada’s Section 56 exemption, a case-by-case exemption program allowing patients at the end of life to access psychedelic therapies legally. Patients who already had a Section 56 exemption, but did not have access to a psychedelic therapist, were also candidates for the program and were enrolled directly. Patients without a Section 56 exemption were supported in applying to Health Canada. Cohorts three and four accessed psilocybin through Health Canada’s Special Access Program, which was adopted in January 2022 to replace the Section 56 exemptions.

BOX 1 **Inclusion/exclusion criteria for psilocybin-assisted therapy.**

**Inclusion criteria:**

Patient must have a terminal diagnosis whereby there is a limited life expectancy (predicted life expectancy less than 2 years).

Ages 19–80 years of age

Male or menopausal female or if childbearing age, not pregnant, using birth control and negative pregnancy test prior to psilocybin administration.

Ambulatory (Palliative performance status 50% or greater)

eGFR >20 mL/min

AST < 3xULN and bilirubin <50 umol/L

Patient has emotional distress that has not successfully responded to other treatments: other treatments failed, patient could not tolerate other treatments, patient is unable to access other treatments, or patient refused other treatments for reasons acceptable to our treatment team.

Patient demonstrates comprehension sufficient for understanding the consent form.

**Exclusion Criteria:**

Pregnancy, Nursing

Currently taking on a regular (e.g., daily) investigational agents, MAO inhibitors, UDG modulators, inhibitors of UGT1A9 and 1A10, aldehyde or alcohol dehydrogenase inhibitors. *Patients who wanted to enrol were given the opportunity to wean off their medication under the direction of their primary care provider or the treating physician*

Current or past history of meeting DSM-5 criteria for (diagnosis must have been confirmed by a qualified psychiatrist or psychologist):

• Schizophrenia;

• Dissociative Disorder

• Psychotic Disorder (unless substance-induced or due to a medical condition);

• Borderline Personality Disorder;

• Bipolar I Disorder;

• Bipolar II Disorder;

• Other psychiatric conditions judged to be incompatible with establishment of rapport or safe exposure to psilocybin.

• ★ Borderline personality disorder, bipolar I disorder and bipolar II disorder may be considered after a psychiatric consult.

• ★ Bipolar I would require more in-depth investigation in relation to the history of manic episodes.

Severity of depression or anxiety symptoms warranting immediate emergent treatment and require immediate referral to community psychiatry.

First degree relatives meet DSM-5 criteria for bipolar disorder or schizophrenia.

Concurrent use of illicit drugs causing ongoing intoxication

Unstable housing

Patients with known sensitivities to psilocybin and or its metabolites or have had significant adverse events after prior psilocybin or other psychedelic use.

Active uncontrolled epilepsy.

Uncontrolled cardiovascular conditions: uncontrolled hypertension, uncontrolled angina, a clinically significant ECG abnormality, TIA in the last 3–6 months, stroke with loss in mental status, peripheral or pulmonary vascular disease with active claudication.

Unstable Insulin-dependent diabetes

Palliative Performance Score of less than 50%

### Consent

Prior to beginning the program, participants were invited to an information session. Participants had a one-to-one session on at least two occasions with the lead physician or lead registered nurse. Patients signed a consent form for enrolment, including an option to allow their de-identified data. All participants consented to the option of including their data in quality improvement initiatives, and for research and publication.

### Intake process

The patients completed intakes with a program physician specializing in palliative care and psilocybin therapy. There was an additional session with a registered nurse specializing in psychedelic therapy and mental health. Data-gathering included: demographics, mental health questionnaires, and short answer responses to specific questions. Participant eligibility was confirmed, with each participant’s supports and resources assessed to predetermine adequate engagement with the therapy structure and the group psilocybin session (Table 1). While patients were encouraged to taper off antidepressants that may compromise the benefit of psilocybin, this was not a requirement. Finally, intentions of joining the program were reviewed to ensure goodness of fit with the program’s intentions.

**Table 1:** Participant demographics.

Demographics	Number of patients
Gender Male/Female	11/14
Age distribution	32–79 years of age
Ethnicity	23 identified White 2 identified part Indigenous
Malignancies	3 metastatic breast cancer
	3 metastatic gynaecological cancers
	2 metastatic malignant melanoma
	1 esophageal cancer
	1 cholangiocarcinoma
	1 pancreatic adenocarcinoma
	3 metastatic lung cancers
	2 primary brain cancers
	3 hematological cancers
	5 metastatic bowel cancers
	1 chondrosarcoma
Undergoing active cancer treatment	17
Previous psilocybin use	5

Four iterative cohorts were completed over the span of 1 year (November 2021–November 2022) with interviews occurring at the end of each cohort. Patients were allowed to withdraw from the study at any point. Patients were all interviewed at one clinic location in Nanaimo, BC using a semi-structured question list, and transcripts were coded and themes captured in an iterative manner. The question list was created from a review of literature and expert opinion. Questions asked included exploration of benefits and challenges to the program, experience with the psilocybin medicine, as well as overall individual experiences and outcomes (Table 2). Interviews were recorded and manually transcribed. Transcriptions were also manually cleaned to remove any identifying information. Data was managed manually on Microsoft Word and Microsoft Excel. The qualitative interviews were organized, and themes were developed in a code book. Two reviewers independently reviewed and coded all data. In the first coding cycle, reviewers identified recurring words, phrases, and meaning units in participant responses. Codes were then compared, combined, renamed, or separated through discussion and re-review of the transcripts. A third independent reviewer reviewed the initial codebook and themes, after which the team iteratively refined the themes by returning to the transcripts and checking whether the candidate themes captured the breadth and nuance of participant accounts. Merriam’s Basic Qualitative Research Approach (31) was used to organize the analysis around participants’ descriptions of meaning, process, and experience in a real-world program. Grounded theory was used pragmatically as an inductive strategy: rather than testing a pre-existing theory, the team allowed categories and relationships among categories to emerge from repeated comparison of participant accounts. Subthemes were further identified and grouped accordingly.

**Table 2:** Semi-structured interview questions.

Questions
Have you had a previous psilocybin assisted therapy experience?
What was your PaT experience like?
Of the above experiences, which format of psilocybin did you prefer?
What PaT-related components of the program could be improved?
Would you be interested in participating in another group PaT session?
What components of the program did you like the most (where the most helpful)?
What tools and practices did you find the most helpful?
What curriculum components of the program could be improved?
What could the program be improved?
How do you feel about the amount of weeks and timing of CoP?
What was your overall impression of the program?
Knowing what you know now, how would you advise your future selves?
What changes have you noticed after you finished the program?

## Results

There were 25 total participants in cohorts one, two, three and four combined. Twenty-one participants (84%) remained with the program from beginning to end. One patient in cohort two and one patient in cohort three passed away during the preparation phase of the program from their underlying cancer prior to receiving psilocybin treatment. One patient in cohort one did not fully engage with preparation and integration sessions; however, they did participate in the scoring and exit interviews. One participant in cohort two did not fully engage with the integration sessions. One participant in cohort three dropped out after the third week prior to the psilocybin session and was lost to follow-up. In total, there were 31 in-person psilocybin medicine sessions provided for 25 participants over the year of the project; six participants repeated the medicine session as alumni within the year. The term PaT is used in this paper to refer to the full therapeutic process, including preparation, psilocybin medicine session, and integration, whereas ‘medicine session’ refers specifically to the day on which psilocybin was administered. Table 3 shows the themes that emerged after transcribing and coding post-program exit surveys, representing 21 of the 25 participants who came through the program.

**Table 3:** Medicine composition, dose, onset, peak, integration.

Medicine	Dose	Onset	Most intense effect	Integration
Dried psilocybe cubensis	Up to 5 grams	25–40 min	1.5–3 h	5–8 h mark
Synthetic psilocybin	25 mg	7–10 min	10 min–2 h	3–4 h mark
Extract of psilocybe cubensis	25 mg	20–30 min	1–3 h	4–6 h mark

The goal of this article is to explore the practicality and participant experience of group administered psilocybin-assisted therapy, rather than studying the efficacy of psilocybin for this patient profile. This notwithstanding, it is important to note factors that seem to mediate efficacy, including the form in which psilocybin was offered, resulting in differences with onset, peak experience, and duration of effect (Table 3). Five of the six alumni participants who had the opportunity to access psilocybin in different forms, reported on the differences in subjective effect (Table 4, Box 2). The participants that had more than one experience with psilocybin (in various formats), tended to prefer whole mushrooms compared to synthetic/extract versions. One shared, “synthetic psilocybin was “more square*” than natural mushrooms. I missed the flow state at end of journey.”* Another noted, “I went deeper on mushrooms [compared to previous synthetic version of mushrooms] …this needs to be there for partners of people with cancer – maybe as couples. Ideally, I’d be scheduled for a session every 6 months.”

**Table 4:** Forms of psilocybin taken (whole mushroom versus synthetic versus extract), how many times for each, and preference in retrospect.

	Order of experience	Preference	“Of the above experiences, which format did you prefer?”
P1	First: synthetic Followed by extract	Extract	“I liked the extract. [2nd sit]. The first time [synthetic] I kept coming out with different worries…would my ostomy bag break… With the extract I was in for the whole time and could not believe we were already done.”
P2	First: whole Followed by extract	Whole	“The mushroom sit was the best. I can revisit it whenever I feel like I’m heading toward a low. The extract was creepy and dark. I had no visuals and the only light I could see was from the two sitters. I got no insight but got a headache. For 2 or 3 weeks after I had an odd, physical sadness and did not even care. I’d never do the extract again.”
P3	First (previous to RTT): whole Followed by synthetic Followed by extract	No discernible preference	“Hard to say. I have no preference. I had nausea with all and all were profound. The synthetic was more intense than extract and went pretty straight up then plateaued for a long time and dropped right off. There was no nice slow re-entry.”
P4	First (previous to RTT): Whole Followed by synthetic Followed by extract	Whole Mushroom	“Mushrooms are best. The extract behaved more like mushrooms than the synthetic. The synthetic was more intense. It went straight up, plateaued a long time, then went straight down with no gentle come down. The extract was like mushrooms at the lower end of the scale. I felt it did not last as long. I would have extract again but maybe a slightly stronger dose.”
P5	First: whole Second: extract	Whole Mushroom	P5:” The mushrooms were the best. With them, I was IN the experience. With the extract, I was watching the experience and when the music paused, the screen went blank. I would take the extract again but liked the mushrooms best.”

BOX 2 **. Participant quotes**

**THEME 1 PaT preparation: prepare well; trust the process; trust the people; feel the feelings**

*Knowing what you know now, how would you advise your future selves?*

**“**To not have any expectations or hopes. Leave preconceived notions behind and you will not have such a let-down. I came in overconfident.”

“Be patient. Things do not have to be 20 miles an hour. It’s OK to slip into the water and enjoy.”

“To make the most of it. To take the time to explore your feelings. Ask as many questions as you want and know you are in a very safe place.”

“Spend time losing the fear. Explore the fear. When the story comes up, practice self inquiry.”

“Make sure you are well rested for the evening sessions. Stay hydrated. Be prepared. Be more settled”

“Be as open and free to speak your true thoughts and listen deeply to what others are saying.”

“Spend time losing the fear. Explore the fear. When the story comes up, practice self inquiry.”

“I looked at what went sideways with the sit in Jamaica. I wasn’t well prepared for it and I learned from that. Being in my group made me feel safe and secure and I knew the space. Pre-session re-building group relationships.”

[alumni] “The immediate afterglow was not as pronounced as the first session. The second session was profound but different. The big lesson was the connection with the body. I took the medicine, then [energy medicine clinician] started doing energy work for my upset stomach. The Energy work was like opening a door and I went under my blanket weeping with my whole body. It felt great after. It went to a place it needed to get to.”

*What was your PaT experience like?*

“It was so good to arrive in my body and get present. I came in with ‘I’ve got some trauma to process - the intensity of this last year. Now it does not touch me, who I am was never threatened.”

“I am a warrior goddess! I was firmly told as much. And I am loved. *zero pain* WOWZA”

“You can feel very sad, alone and forgotten about. But not now. I have friendlier internal dialogue and my biggest takeaway was listening”

**THEME 2 Gaining as positive outlook**

*Subtheme 2a distancing from negativity*

*What changes have you noticed after you finished the program?*

“I have lost friends who were trying to “fix” me. I’ve learned to be grateful for every day. Separating myself from negative people. Friends say I look different. Serene and calm. I am loving in the moment.”

“I was already on the path, but this gave me a boost and made me aware of what’s really important: Time with friends and family.”

*What was your PaT experience like?*

[alumni] “I felt there was more work for me to do with the psilocybin. I was on the edge of knowing the question I needed to ask. The answer came like a whack to the head. I had unresolved anger with my mother who is passed. I wanted her approval. During the sit, I was under water and bashing my head over and over on a cement jetty. I was told, ‘You aren’t going to get past that until you inwards for approval’. Suddenly I was freely swimming. It was amazing!”

*Subtheme 2b improvements in mood*

*What changes have you noticed after you finished the program?*

“No panic attacks for over 2 months! “

“I do not cry as heavy. I sing more and I think I carry a tune better. I feel lighter. Music excites me more. I enjoy the positive and bright energy outdoors. My mind is amazing.”

“I’m having less negativity.”

“I’m crying less and doing more. I’m more positive and I’m going all out for Christmas. I’m telling everyone that psilocybin changes the pathways in your brain.”

“I get really big lows but I’m processing them rather than let it build up. Since the first sit I have advanced so fast. To come back out and have people back in my life that I had cut most people out.”

“My husband is the best judge and he sees the difference. I am not as deeply saddened.”

*What was your PaT experience like?*

“My experience was peaceful, restful. I can see how the music helps pull us in and out of stuff.”

“The medicine is the key that unlocks the door to the journey.”

[alumni] “Last time, I came out knowing I was fundamentally different – not the same person. Like my DNA was restructured and I was made whole…but I was still carrying weight. This time, I came out much lighter.”

*Subtheme 2c honesty and authenticity*

“Profound change. Potentially everything changes. If I choose to be aware I can be.”

“More honesty with self and people I love. It changed me to the core of myself. I’m firm in making boundaries. I’m not there for your drama.”

“It changed me to the core of myself.”

“I was shocked in a good way seeing the changes in everyone.”

“I do not know if I would have been as honest. Honesty is the only way I know how to combat fear.

“Here’s who I am and here’s why I’m here.”

Subtheme 2d A greater focus on how to navigate death, pain and loss

“I’m less worried about death – more at peace. I’m less stressed out. Not thinking about it all the time.”

“could have been more focused on facing death and how to live an extra day as happy and healthy as possible with mental health”

“[I Liked] making connections with a group of people who have had similar experiences, thoughts and feelings living with cancer.”

“I felt wretched the next day and wish I knew how to manage. We did not talk about our diagnosis and how we feel about it…”

“Battle. Connection to tumour. Mourning for loss. Goodbye tumour. My brain is rewiring.”

**THEME 3 The community of practice (CoP) as the primary conduit for connection and regulation**

*What components of the program did you like the most (where the most helpful)?*

“Compassionate communication, self-compassion, and community were all helpful…”

“meditating…helps with anxiety”

“Farting around with RAIN. Allowing my feelings. Reminders to stop judging to shorten my cruddy moods with deep breaths.”

“The awkwardness of zoom was a problem. Face to face would have made it better.”

Felt like we were all in this together.”

“The group fed itself.”

“Group made it so powerful.”

“The identity I found in the group was so important.”

“[This group process was] very important…”

“It has been outstanding for me – to be in a group with such a special tender dilemma in our lives.”

“The benefit of the whole package has been huge – more than just the mushrooms. I would pay to continue being in group without the mushrooms.”

“Very important. I felt connected, accepted and able to talk to people going through the same things. People say what you are thinking because they have the same struggles.”

[alumni] “Going to the meetings was a priority for me which is different than planning to get together with each other.”

*What tools and practices did you find the most helpful?*

[alumni] “I had anticipatory distress about meeting new people, but I felt very supported and included in the group. It helped me to hear how different everyone’s experiences were and helped me let go of my expectations [that this sit would be as good as the first one]. I experienced losing my self-consciousness with the group because they were so respectful and safe.”

*What was your PaT experience like?*

“Not what I expected. At first I wasn’t convinced I was having any effects at all from the medicine, but the encounter with the snake changed that. I did not see any fanciful visions. “I felt a deep connection to the energy in the room. It was profound.”

**THEME 5 Challenges and benefits**

*Subtheme 5a overall benefits*

*What was your overall impression of the program?*

“I’m so incredibly thankful. The most profound, beautiful experience.”

“I thought it was beautifully done.”

“The whole program was well done.”

“Glad to be part of this and sometimes it was hard to hear sad stories.”

“Profound”

“. This is cutting edge. I want to advocate for psychedelics.”

“Very important.”

“well thought out and well put together.”

“I felt a generous warmth that inspired courage and the thread of humanity was respected. I do not regret my experiences…”

“More honesty with self and people I love. It changed me to the core of myself.”

“I enjoyed it! We knew every week that was our time.”

“It was an honour to be part of it. To help me and help others.”

*Subtheme 5b challenges*

*What PaT-related components of the program could be improved?*

“I think that it is extremely important to give individuals the option to consume psilocybin in private… Sharing your experiences with the group before and after is an obvious staple, however I do not see how it is beneficial to me, in the middle of my own journey, to hear 5 other participants chatting/snoring/puking/crying… the distraction of watching people endlessly enter and exit a room was turbocharging my anxiety. Although I can see how it would be a logistical nightmare having 10 private rooms for 10 participants, the option must exist.”

“More time for the mushroom journey (I particularly felt rushed at the end of that day)”

*Would you be interested in participating in another group PaT session?*

“I do not see the need. I’m interested but do not see the need.”

“I would totally do it – like a maintenance thing. More exploring in case there’s more to explore.”

“Maybe somewhere with more control…like my home.”

*Subtheme 5c impacts of the therapy team*

“…team made everyone feel comfortable and safe. Very professional.”

“Feeling completely supported by the group of medical people and carers who also shared with the group their life experiences…”

“Loved RTT guidance, acceptance, and openness.”

“Very experienced team with psilocybin and ketamine”

“Perhaps communicate and honour team needs. You soak up all the tears.”

“More information about specialists on the team and their area of expertise – how they could help us.”

*Note*: Quotes included are representing examples of similar quotes in the subtheme area.

Overall themes derived from the qualitative interviews revolved around the intentions and therapy goals set out by participants. Analysis of quotes from participant interview content revealed themes of: trust and attunement with the innate self, gaining capacity and perspective, authenticity and honesty, connection and regulation with others, and navigating loss and death (Table 4). The theme ‘trust and attunement with the innate self’ links two related aspects of participants’ accounts: first, trust in the therapeutic process, team, group, and unfolding medicine experience; and second, increased ability to listen to internal bodily, emotional, and intuitive cues rather than relying primarily on external control or reassurance.

When participants were asked how they would advise their future selves, ten spoke to the importance of managing expectations and trusting the process. One participant shared, “trust the process. Be open. Be vulnerable. Be 100% honest. Be dirty. Don’t gloss it over. The only way you are truly going to get what you need is if you lay it all out there.” Six participants commented on the importance of making it their own, asking lots of questions to cultivate understanding, ensuring they are as informed as possible before they begin, and feeling the emotions that arise as an important (even imperative) part of the preparation process. Many individuals commented on the subtheme of strength in oneself nothing for example, “I felt the strength of women, I felt the strength, the depth of grace and so much healing. Experiencing so much power of healing within myself. I also felt the strength of the people holding the space so I could feel all this.”

There were 32 mentions of various regulation practices that participants used mitigate stress activation in their bodies, enabling a greater ability to experience relational connection. Sixteen participants spoke to their appreciation of the somatic energy modalities (primarily tapping) as their preferred regulation practice. The ability for self-regulation was also noted to impact other domains of life. One participant shares, “it has affected me, and my focus has altered. I’ve got more focus on compassion that I haven’t given myself or others and being more forgiving and less demanding without giving it all away*”.*

Sixteen participants noticed being less attached to external noise. Nineteen participants commented on an increased perspective, positivity, and peace. One participant describes her shift in priorities as “[it] gave me a “booster shot” *to help decrease the “noise” in my world. I’m good at recognizing this and restoring homeostasis.”* Another commented that, “I am better able to distance myself from everyone else’s problem. I don’t need to fix everything.”

Seven individuals commented on the feeling of being lighter and experiencing less stress. In the realm of feeling a new lightness, some participants commented on objective indicators for improvement in their mood disorder while others noted subjective feelings of positivity and enjoyment. One alumni notes, “I haven’t taken the Ativans for a while. I have some depression still around finances and my ex but [am] less anxious.” While another shares, “I am less bitchy and more aware of what’s going on in me. I feel more space to respond. I am a work in progress and feel the changes. My emotions are not stable and my answers to these questions are different on different days. I can feel the change. I know what makes me happy and I’m more selective of what I want.”

Seven noticed more honesty and authenticity. One participant shares, “a door opens to honesty. I know myself right down to the ground. I have the increased ability to thrive by walking with others.*”*

Eight participants spoke to an increased openness on topics such as death, pain and loss. One participant shares, “I don’t have the message in my head that I’m dying of cancer. You can change your mind. My mind would go to the negative but not anymore.” Another notes that, “I am living this life and choosing to be alive, it’s impossibly hard. It was a real, visceral experience.”

### Facilitating connection to community

This finding was particularly relevant to the study’s focus on a hybrid model: the virtual CoP appeared to establish continuity, familiarity, and peer witnessing before the in-person medicine session, while the medicine session intensified the sense of shared experience for many participants. Participants mentioned the CoP as a support mechanism in their healing process in 22 quoted statements with multiple accounts of the group process being the most helpful part of PaT.

“The connection built between the participants in the weekly meetings was very meaningful. Hearing others open up about their struggles with their diagnosis was important for me to be able to share my feelings.”

Another noted that, “this was way beyond what I expected. It couldn’t have happened without this group and how it all came together. I could go today, but it wouldn’t matter, it was so profound…”

Seven individuals spoke specifically feeling more connected, loved and/or accepting after the PaT session and four noticed subtle or no changes in relational capacity post-program.

Another participant captured the difference between solitary and group-based therapy by stating that the group “made it so powerful,” while another said, “the identity I found in the group was so important.” These comments suggest that, for many participants, the group was not simply a logistical structure but an active therapeutic ingredient.

One participant shared, “*Be as open and free to speak your true thoughts and listen deeply to what others are saying.”* Multiple participants commented on the importance of developing a sense of safety with group members stating that, “they feel like family…other peoples’ perspectives made me feel less lonely. I felt supported and safe with the shared experience. The thought of doing it alone makes me sad.”

Participants also noted a connection to ancestors and perceived community noting, “the experience was profoundly beautiful. A lot of ‘goodbyes’ to say - like to my mother. I felt my ancestors so deeply, but also the ancestors of the elders from this place were here and I felt the strength.*”*

Fourteen participants commented broadly about meaningful experiences and positive outcomes from the PaT session. Seven spoke to challenges that arose in the PaT session. For instance, three did not like the music, with one individual stating, “[the] music was overwhelming for some and we couldn’t get away from it.” Three participants did not like the physical environment of the psilocybin session noting that there was a noisy fan and not enough natural light in the room. Two spoke to abdominal discomfort during or after the session; two would have preferred a private (instead of group) PaT experience.

When asked if participants would be interested in participating in another group PaT session, 16 said yes; one would prefer a private sit, one did not feel the need, others did not feel a pull either way, one felt multiple sessions should be offered as a standard.

Fourteen participants spoke to the constitution and interpersonal capacity of the team as a major contributor in their positive experience of the program. Descriptions provided by individuals also highlighted not only the professional nature of the therapists, but the continued warmth and relational capacity echoed in a other themes. One participant noted that the best part of the program was, “the incredible consistent care, protection, and guidance the team presented/bestowed upon the participants every step of the way…”

## Discussion

In the spirit of the iterative learning approach of RTT as a continuous learning system, the discussion of themes was explored with practice implications in mind.

Reflections from participants are consistent with goals of therapy documented in PaT literature, notably Danforth’s (30) PaT preparation guidance. Key elements include establishing intentions but surrendering these intentions while under the influence of psilocybin, in favour of the felt sense experience, or to one’s “inner healing intelligence”. While there needs to be adequate content to optimally support their experience, sharing information that may not be relevant can lead to unnecessary priming (32). Therefore, therapy materials should promote a sense of predictability and confidence, while also mitigating unnecessary suggestion, or priming of expectation. The development of intentions that encourage trust in one’s inner healing intelligence to inform the *process* can help mitigate disappointments from expectations focussed on a desired *outcome*. Attendance requirements are likely an important safety mechanism, ensuring the relational alliances are well formed before participants embark on a group PaT experience. This further promotes trust and relational safety and ensures from a pragmatic standpoint, that participants are present when preparation content is delivered.

In addition, offering a variety of regulation practices enable participants to choose what works best for them; however, too many choices can lead to feeling overwhelmed. Jerath et al. (31) speaks to the effectiveness of breathing techniques for the treatment of anxiety, with fourteen of our participants benefiting from the practice of various breathing exercises over the course of the program. In a systematic review, tapping/Emotional Freedom Techniques (EFT) was found to be effective against depression (33).

### Gaining a positive outlook on life

Results suggest that for the majority of those experiencing terminal health conditions, psilocybin-assisted therapy can promote positive changes in one’s outlook and experience of life. Swift et al. (34) found similar results in their study, including increased positivity in outlook, feelings of peace, and honesty. Participant comments also indicated a desire for more explicit preparation around living with terminal illness, including one participant’s suggestion that the program could be “more focused on facing death and how to live an extra day as happy and healthy as possible with mental health.” Future versions of the curriculum may benefit from dedicated modules on diagnosis-related grief, anticipatory loss, and practical integration of insights about death into remaining life.

Approaching themes of death and dying more explicitly with those facing end of life are imperative in PaT. MacKenzie & Lasota (35) speak to how conversations about death and dying are a crucial part of all end of life care. Barriers to carrying out these conversations relate to deficiencies in training, lack of confidence, cultural differences, and limited time. While the clinical team in RTT is adept at such conversations, implementation challenges relate to time limitations and concerns of participants being overwhelmed with too much content. Finding an optimal balance would be a focus for further research.

### Facilitating connection to community

Intentional, and structured virtual CoPs can be an effective tool to promote a sense of community and connection. In Andersen et al. (36) systematic review, findings demonstrate how social relationships influence mental health, and speak to the potential for digital interventions that can promote the development and strengthening of relationships despite their requirement for focus on quality and content for efficacy.

There is an abundance of literature that speaks to the impacts of set and setting on therapeutic outcomes. Hartogsohn (37) compares set and setting theory (prescriptive for therapists) to placebo theory (descriptive of mechanism), both of which focus on the environmental (non-biologic) factors that can inform therapeutic outcomes. Naturalistic settings with natural light and exposure to nature is ideal. In the present program, participant feedback about music, fan noise, natural light, room traffic, and availability of private space highlights why detailed reporting of setting is especially important for group PaT, where one participant’s movement, distress, or somatic symptoms may become part of another participant’s setting.

While an individual session may be preferred by some, the abundance of positive feedback related to peer support suggests that the group framework offered benefits not readily available in a solo model. The individual model may offer greater privacy, fewer sensory disruptions, more personalized music and pacing, and reduced exposure to other participants’ distress. In contrast, the group model offers peer witnessing, normalization, shared courage, and opportunities to receive unconditional positive regard and compassionate reflection from people facing similar challenges. The virtual CoP model enabled patients to join from more remote areas, only requiring travel for the psilocybin-assisted therapy session; however, virtual delivery also introduced limitations, including the awkwardness of Zoom and reduced embodied presence compared with in-person preparation. Compared to the cost of individual sessions, group sessions are more efficient, potentially expanding access with reduced costs and increased therapist productivity. The findings therefore do not suggest that group PaT should replace individual PaT, but rather that a hybrid model may be a scalable and therapeutically distinct option for patients who value community and can tolerate the sensory and interpersonal demands of group dosing.

### Identified program benefits and challenges

The two participants who preferred an individual sit appear to identify a key boundary condition for this model: group therapy may be most suitable when participants value peer connection and can remain regulated in the presence of others’ sounds, movement, and emotional expression. Screening and preparation should therefore include discussion of sensory sensitivity, need for privacy, prior group experience, and willingness to encounter other participants’ distress during the medicine session.

With completion rates of over 80% in a population that is navigating complex medical and emotional challenges, this iterative program development pilot supports the feasibility of group psilocybin sessions within group therapy using a CoP model. The amount of feedback pertaining to the benefits of peer support suggests that this format may have distinct advantages to the traditional solo sessions for patients with distress related to a terminal diagnosis. Although disease progression and prognosis is difficult to predict in patients with terminal illness, a more streamlined national approach of access to PaT could possibly allow more patients to participate before their illness progresses.

This program additionally meets one of the largest barriers to access in PaT-cost. By using a group model, the cost of the program and ongoing support is projected to be significantly less than for a MAPS-type dyad model (21). Beyond cost, finding a physician trained in psychedelic medicines, who is also able to support an application for special access to psilocybin, and/or accessing an eligible clinical trial are further challenges.

Slavin-Mulford (38) reviewed multiple research studies and found that the interpersonal capacities of the therapist can have a greater impact than the treatment it is supporting. Interestingly, interpersonal skills appeared to be either inherent, or acquired through life experience, and independent of formal education. In the development of therapy teams, assuming basic credentials are attained, the interpersonal skills of the team should be a primary focus Given the toll on healthcare providers amid a mental health crisis, therapists also need to be adequately supported within a culture of compassion and safety. This supports their own resilience and capacity for maintaining a healing therapeutic presence (39).

### Limitations

Patient selection in this study was based on individuals who were able to obtain permission to consume psilocybin from Health Canada (Section 56 exemption or Special Access program). This constitutes a significant selection bias, as only those patients with the ability and resources to navigate the regulations were able to enrol in the program. Furthermore, five patients with Section 56 exemptions for psilocybin use had availed themselves of psilocybin treatment with private therapists within a 12-month period prior to participating in the program. This could have had a confounding effect on their response to the program. Although the results of this study are helpful in shedding light on the qualitative impacts of group PaT on a specific vulnerable population, this study was not constructed as a clinical trial and no randomization or control group was available. Further research on group administered PaT with virtual group CoPs would be helpful in forming more generalizable conclusions.

This innovative pilot program represents the first of its kind in North America, demonstrating that PaT can be safely and effectively provided in a group setting, supported by an experienced multidisciplinary team, and optimized through a virtual CoP framework. The study suggests that group PaT differs from individual PaT not merely in efficiency, but in therapeutic mechanism: many participants described peer witnessing, shared vulnerability, and community belonging as central to the experience. At the same time, participant critiques of music, room conditions, group noise, movement, vomiting, and preference for private dosing indicate that group administration also creates setting-related risks that require careful design and reporting. While results were generally positive, there continues to be a paucity of research informing hybrid group therapy models. The results of this qualitative study offer important considerations in effective group PaT as psychedelic treatments continue to gain momentum in North America, particularly the need to balance scalable community-based access with participant choice, sensory control, and sufficient preparation for both group and individual needs.

## Data Availability

The original contributions presented in the study are included in the article/supplementary material, further inquiries can be directed to the corresponding authors.

## References

[1] Mitchell AJ Chan M Bhatti H Halton M Grassi L Johansen C . Prevalence of depression, anxiety, and adjustment disorder in oncological, haematological, and palliative-care settings: a meta-analysis of 94 interview-based studies. Lancet Oncol. (2011) 12:160–74. doi: 10.1016/S1470-2045(11)70002-X, 21251875

[2] Skarstein J Aass N Fosså SD Skovlund E Dahl AA. Anxiety and depression in cancer patients: relation between the hospital anxiety and depression scale and the European Organization for Research and treatment of cancer core quality of life questionnaire. J Psychosom Res. (2000) 49:27–34. doi: 10.1016/s0022-3999(00)00080-5, 11053601

[3] Grassi L Caruso R Hammelef K Nanni MG Riba M. Efficacy and safety of pharmacotherapy in cancer-related psychiatric disorders across the trajectory of cancer care: a review. Int Rev Psychiatry. (2014) 26:44–62. doi: 10.3109/09540261.2013.842542, 24716500

[4] Ostuzzi G Matcham F Dauchy S Barbui C Hotopf M. Antidepressants for the treatment of depression in people with cancer. Cochrane Libr. (2018) 2018:CD011006–74. doi: 10.1002/14651858.CD011006.pub3, 29683474 PMC6494588

[5] Agin-Liebes GI Malone T Yalch MM Mennenga SE Linnae Ponté K Guss J . Long term follow-up of psilocybin-assisted psychotherapy for psychiatric and existential distress in patients with life-threatening cancer. J Psychopharmacol. (2020) 34:155–66. doi: 10.1177/0269881119897615, 31916890

[6] Ross S Bossis A Guss J Agin-Liebes G Malone T Cohen B . Rapid and sustained symptom reduction following psilocybin treatment for anxiety and depression in patients with life-threatening cancer: a randomized control trial. J Psychopharmacol. (2016) 30:1165–80. doi: 10.1177/0269881116675512, 27909164 PMC5367551

[7] Ross S Agin-Liebes G Lo S Zeifman RJ Ghazal L Benville J . Acute and sustained reductions in loss of meaning and suicidal ideation following psilocybin-assisted psychotherapy for psychiatric and existential distress in life-threatening cancer. ACS Pharmacol Transl Sci. (2021) 4:553–62. doi: 10.1021/acsptsci.1c00020, 33860185 PMC8033770

[8] Griffiths RR Johnson MW Carducci MA Umbricht A Richards WA Richards BD . Psilocybin produces substantial and sustained decreases in depression and anxiety in patients with life-threatening cancer: a randomized double-blind trial. J Psychopharmacol. (2016) 30:1181–97. doi: 10.1177/0269881116675513, 27909165 PMC5367557

[9] Lowe H Toyang N Steele B Valentine H Grant J Ali A. The therapeutic potential of psilocybin. Molecules. (2021) 26:2948–81. doi: 10.3390/molecules26102948, 34063505 PMC8156539

[10] Johnson MW Griffiths RR Hendricks PS Henningfield JE. The abuse potential of medical psilocybin according to the 8 factors of the controlled substances act. Neuropharmacology. (2018) 142:143–66. doi: 10.1016/j.neuropharm.2018.05.012, 29753748 PMC6791528

[11] Glennon RA Titeler M McKenney JD. Evidence for 5-HT2 involvement in the mechanism of action of hallucinogenic agents. Life Sci. (1984) 35:2505–11. doi: 10.1016/0024-3205(84)90436-3, 6513725

[12] Carhart-Harris RL Leech R Hellyer PJ Shanahan M Feilding A Tagliazucchi E . The entropic brain: a theory of conscious states informed by neuroimaging research with psychedelic drugs. Front Hum Neurosci. (2014) 8:1–22. doi: 10.3389/fnhum.2014.00020, 24550805 PMC3909994

[13] Stamets P. Psilocybin Mushrooms of the World. Berkeley: 10 Speed Press (1996). p. 10–2.

[14] Schultes RE Hofmann A Rӓtsch C. Plants of the Gods: Their Sacred, Healing, and Hallucinogenic Powers. Rochester, VT: Healing Arts Press (2001).

[15] Anderson BT Danforth A Daroff R Stauffer C Ekman E Agin-Liebes G . Psilocybin-assisted group therapy for demoralized older long-term AIDS survivor men: an open-label safety and feasibility pilot study. EClinicalMedicine. (2020) 27:1–11. doi: 10.1016/j.eclinm.2020.100538, 33150319 PMC7599297

[16] Agin-Liebes G Ekman E Anderson B Malloy M Haas A Woolley J. Participant reports of mindfulness, posttraumatic growth, and social connectedness in psilocybin-assisted group therapy: an interpretive phenomenological analysis. J Humanist Psychol. (2021) 64:564–91. doi: 10.1177/00221678211022949

[17] Griffiths RR Johnson MW Richards WA Richards BD Jesse R MacLean KA . Psilocybin-occasioned mystical-type experience in combination with meditation and other spiritual practices produces enduring positive changes in psychological functioning and in trait measures of prosocial attitudes and behaviors. J Psychopharmacol. (2018) 32:49–69. doi: 10.1177/0269881117731279, 29020861 PMC5772431

[18] Grof S Grof C. Holotropic Breathwork: a New Approach to Self-Exploration and Therapy. Albany: State University of New York Press (2010).

[19] Spiegel D Classen C. Group Therapy for Cancer Patients: a Research-Based Handbook of Psychosocial Care. New York: Basic Books (2000).

[20] Spiegel D Yalom ID. A support group for dying patients. Int J Group Psychother. (1978) 28:233–45. doi: 10.1080/00207284.1978.11491609, 631956

[21] Spiegel D Glafkides C. Effects of group confrontation with death and dying. Int J Group Psychother. (1983) 33:433–47. doi: 10.1080/00207284.1983.11491344, 6642804

[22] Leszca M Goodwin P. The rationale and foundations of group psychotherapy for women with metastatic breast cancer. Int J Group Psychother. (1998) 48:245–73. doi: 10.1080/00207284.1998.11491538, 9563240

[23] Classen C Butler LD Koopman C Miller E DiMiceli S Giese-Davis J . Supportive-expressive group therapy and distress in patients with metastatic breast cancer: a randomized clinical intervention trial. Arch Gen Psychiatry. (2001) 58:494–501. doi: 10.1001/archpsyc.58.5.494, 11343530

[24] Trope A Anderson BT Hooker AR Glick G Stauffer C Woolley JD. Psychedelic-assisted group therapy: a systematic review. J Psychoactive Drugs. (2019) 51:174–88. doi: 10.1080/02791072.2019.1593559 30950777 PMC6650145

[25] Agrawal M Emanuel E Richards B Richards W Roddy K. Thambi P assessment of psilocybin therapy for patients with Cancer and major depression disorder. JAMA Oncology. (2023) 9:864–6. doi: 10.1001/jamaoncol.2023.0351, 37052904 PMC10102915

[26] Dames SS. A study of the interplay between new graduate life experience, context, and the experience of stress in the workplace: exploring factors towards self-actualizing as a Novice Nurse [dissertation on the internet]. Calgary (AB): University of Calgary; (2018). Available online at: https://prism.ucalgary.ca/bitstream/handle/1880/106516/ucalgary_2018_dames_shannon.pdf?sequence=1&isAllowed=y (Accessed February 21, 2022)

[27] Dames S Kryskow P Watler C. A cohort-based case report: the impact of ketamine-assisted therapy embedded in a community of practice framework for healthcare providers with PTSD and depression. Front Psych. (2022) 12:1–7. doi: 10.3389/fpsyt.2021.803279, 35095617 PMC8790057

[28] Dames S. Root Strength: a Health and Care Professionals’ Guide to Minimizing Stress and Maximizing Thriving. Cambridge MA: Elsevier Publishing (2022).

[29] MAPS Public Benefit Corporation. Protocol MAPP1 Amendment 4 Version 1. [Protocol synopsis on the Internet]. Santa Cruz, CA: Multidisciplinary Association for Psychedelic Studies (MAPS) (2020).

[30] Danforth A. Focusing-oriented psychotherapy as a supplement to preparation for psychedelic therapy. J Transpers Psychol. (2009) 41:151–60.

[31] Jerath R Crawford MW Barnes VA Harden K. Self-regulation of breathing as a primary treatment for anxiety. Appl Psychophysiol Biofeedback. (2015) 40:107–15. doi: 10.1007/s10484-015-9279-8, 25869930

[32] Johnson MW Richards WA Griffiths RR. Human hallucinogen research: guidelines for safety. J Psychopharmacol. (2008) 22:603–20. doi: 10.1177/0269881108093587, 18593734 PMC3056407

[33] Nelms JA Castel L. A systematic review and meta-analysis of randomized and nonrandomized trials of clinical emotional freedom techniques (EFT) for the treatment of depression. Explore. (2016) 12:416–26. doi: 10.1016/j.explore.2016.08.001, 27843054

[34] Swift TC Belser AB Agin-Liebes G Devenot N Terrana S Friedman HL . Cancer at the dinner table: experiences of psilocybin-assisted psychotherapy for the treatment of cancer-related existential distress. J Humanist Psychol. (2017) 57:488–519. doi: 10.1177/0022167817715966

[35] MacKenzie AR Lasota M. Bringing life to death: the need for honest, compassionate, and effective end-of-life conversations. Am Soc Clin Oncol Educ Book. (2020) 40:1–9. doi: 10.1200/EDBK_279767, 32207670

[36] Andersen LM Rasmussen AN Reavley NJ Bøggild H Overgaard C. The social route to mental health: a systematic review and synthesis of theories linking social relationships to mental health to inform interventions. SSM Mental Health. (2021) 1:100042. doi: 10.1016/j.ssmmh.2021.100042, 38826717

[37] Hartogsohn I. Set and setting, psychedelics and the placebo response: an extra-pharmacological perspective on psychopharmacology. J Psychopharmacol. (2016) 30:1259–67. doi: 10.1177/0269881116677852, 27852960

[38] Slavin-Mulford J. Therapist characteristics that impact outcome. Society for the advancement of Psychotherapy; (2016). Available online at: https://societyforpsychotherapy.org/therapist-characteristics-that-impact-outcome/ (Accessed February 21, 2022)

[39] Dames S Watler C Kryskow P Allard P Gagnon M Tsang V. W. L. Psychedelic-assisted therapy training: Firsthand experience of non-ordinary states of consciousness in the development of competence. Psychedelic Medicine. (2024). doi: 10.1089/psymed.2023.0004, 40051685 PMC11658659

